# Comparative efficacy and safety of traditional Chinese patent medicine in the treatment of *Mycoplasma pneumoniae* pneumonia in children: A protocol for systematic review and meta-analysis

**DOI:** 10.1097/MD.0000000000023747

**Published:** 2020-12-18

**Authors:** Hongan He, Xiao Wang, Yanyan Xiao, Jialin Zheng, Jinjuan Wang, Baoqing Zhang

**Affiliations:** a The First College of Clinical Medicine, Shandong University of Traditional Chinese Medicine; b The Affiliated Hospital of Shandong University of Traditional Chinese Medicine, Jinan, Shandong Province, China.

**Keywords:** *Mycoplasma pneumoniae* pneumonia in children, network meta-analysis, protocol, traditional Chinese patent medicine

## Abstract

**Background:**

*Mycoplasma pneumoniae* pneumonia (MPP) is a common respiratory disease in children. Its incidence rate is increasing year by year. The drug resistance rate of macrolide antibiotics and other conventional treatment methods is higher, and there are limitations in clinical application. Traditional Chinese patent medicine (TCPM) is a powerful weapon to treat this disease. At present, there is no comparison of the safety and effectiveness of multiple TCPMs in the treatment of MPP in children. Therefore, we take the method of network meta-analysis to systematically compare the efficacy of various TCPMs in the treatment of this disease.

**Methods:**

We will conduct comprehensive searches of Cochrane Library, PubMed, Web of Science, Clinical Trials, China National Knowledge Infrastructure, Chinese Scientific Journals Database, Chinese BioMedical Literature, Wanfang Database, and other electronic databases. The time frame is set from the establishment of the database to October 2020. All randomized controlled trials that meet the inclusion criteria will be included in this study. The 2 researchers will independently screen the literature according to the inclusion criteria, extract the data, and assess the bias risk of the included study. We will evaluate all the obtained data and evidence through Bayesian network meta-analysis, and use Stata 15.0 to process and analyze the data.

**Results:**

Through this study, we will evaluate the efficacy and safety of a variety of TCPMs for the treatment of MPP in children.

**Conclusion:**

The purpose of this study is to provide a strong reference for clinical application of TCPMs in the treatment of MPP in children, and to provide an important basis for clinicians to make correct judgments and put forward accurate treatment plans.

**Ethics and dissemination:**

This review does not involve any human or animal experiments and therefore does not require ethical approval.

**INPLASY registration number:**

INPLASY 2020100108

## 1 Introduction

*Mycoplasma pneumoniae* (MP) is a common pathogen of community-acquired pneumonia (CAP) in children. In addition to causing primary atypical pneumonia, we can also see bronchitis and other respiratory tract infectious diseases, clinical symptoms of digestive system, cardiovascular system and nervous system.^[1–3]^ The main clinical manifestations of *M. pneumoniae* pneumonia (MPP) are fever, persistent dry cough, and less sputum. The course of the disease can last for 2 weeks or even longer. It is more common in school-age children, accounting for about 10% to 40% of CAP. It is easily spread in communities and institutions. During the epidemic of MP, the infection rate can reach 30% to 50%.^[4–7]^ At present, macrolide antibiotics are the first-line drugs for the treatment of MP infection. However, long-term use of these drugs will easily change the morphology, structure, metabolism, and other aspects of MP, increasing of clinical drug resistance rate, which makes the treatment of MP infection more difficult.^[8]^ At the same time, due to MP resistance, mixed infection, and immune damage, there are more and more clinical cases of refractory *M. pneumoniae* pneumonia (RMPP) and severe *M. pneumoniae* pneumonia (SMPP). The disease progresses rapidly, and severe cases may have pulmonary complications such as pleural effusion, pulmonary necrosis, and extrapulmonary manifestations such as myocarditis, encephalitis, hepatitis, and even cause multiple organ failure. Some cases can also develop into acute respiratory distress syndrome, necrotizing pneumonia, etc, which seriously endanger children's life and health.^[9,10]^

This disease is mainly caused by MP infection, and its pathogenesis is complex. Previously, the disease was considered as a self-limited disease with mild symptoms. With many experimental and clinical research findings, MP adheres to the surface of epithelial cells of the respiratory tract of the host and produces toxins such as hydrogen peroxide and respiratory distress syndrome, which results in epithelial cell injury, weakening ciliary movement, and inducing cell apoptosis.^[11,12]^ Also, the host immune response may be involved in the pathogenesis of MPP, and there is a certain degree of immune dysfunction during the pathogenesis of MPP.^[13,14]^ The levels of IgG, IgM, and IgA were high in the acute and convalescent stages of MPP. Moreover, the imbalance of Th1 and Th2 cells existed in MPP children, and the dynamic balance of the cytokine network in the body was destroyed, thereby mediating the immune response. At the same time, due to MP antigen has the same antigen structure as the brain, heart, liver, kidney, and smooth muscle of the human body, resulting in MP cannot be effectively immune cleared by host cells. Instead, it can stimulate the body to produce the corresponding antibody, cause cross immune reaction, appear RMPP, SMPP, even encephalitis, myocarditis, hepatitis, glomerulonephritis, autoimmune hemolytic anemia, and other extrapulmonary manifestations.^[15,16]^

There are various treatment methods for MPP in children, and corresponding treatment measures will be taken according to different symptoms, severity of disease, and complications. It mainly includes drug therapy, fiberoptic bronchoscopy intervention, and the combination of drugs and interventional therapy.^[17]^ The therapeutic drugs include macrolides, fluoroquinolones, tetracyclines, and glucocorticoids.^[18]^ Drug therapy mainly plays a therapeutic role by inhibiting the synthesis of MP protein and DNA. However, tetracycline antibiotics can cause yellowing of teeth, enamel hypoplasia, gastrointestinal irritation, hepatotoxicity, and other side effects, which are forbidden to children under 8 years old; quinolones can cause cartilage and joint damage and affect children's bone development;^[19,20]^ macrolides have a resistance rate of up to 90% in some epidemic years,^[21]^ the patients with macrolide resistant MP infection had longer fever duration, hospitalization time, and antibiotic treatment course, which increased the difficulty and cost of treatment.^[22]^ Immunosuppressive agents such as glucocorticoid or immunoglobulin are needed to effectively curb the disease progression. However, there is no consensus on the exact starting time, dosage or duration of immunomodulators.^[23,24]^

Traditional Chinese patent medicine (TCPM) is a kind of traditional Chinese medicine product which is made of Chinese medicine as raw material, in order to prevent and treat diseases according to the prescribed prescription and preparation technology. It has the characteristics of stable property, easy to carry and store. It plays an important role in improving clinical symptoms, preventing disease progression, and reducing adverse reactions. Clinical use of TCPM in the treatment of children with MPP has a good effect, many studies have confirmed this point. The commonly used TCMPs include Xiao’er Xiaoji Zhike Oral Liquid, Pudilan Xiaoyan Oral Liquid, Lianhuaqingwen Granules, Xiaoer Feire Kechuan oral Liquid, etc. Because there are many varieties of TCPMs in the treatment of MPP in children, the comprehensive comparison of different varieties cannot be reflected by traditional meta-analysis. This network meta-analysis protocol will provide clinicians with comprehensive and reliable summary evidence by comparing the efficacy and safety of different TCPMs in the treatment of MPP in children.

## 2 Materials and methods

We will conduct this study in strict accordance with PRISMA-P^[25]^specification.

### 2.1 Study registration

The network meta-analysis is registered with the International Registration System Review and Meta-analysis Research Platform (INPLASY) under the registration number of INPLASY 20201001008.

### 2.2 Inclusion criteria

#### 2.2.1 Type of study

We will include all randomized controlled trials (RCTs) studies published in Chinese or English on the use of TCPMs in the treatment of MPP in children. Still, we need to exclude studies that are not RCT, including meta-analysis, non-clinical studies, lack of data, or poorly designed studies.

#### 2.2.2 Participants

The diagnosis of MPP in children will follow the guidelines for CAP in children.^[26]^ Children aged 1 to 15 years who have been diagnosed with MPP will be included. There are no restrictions on the child's race, gender, or disease severity and course of the disease.

#### 2.2.3 Interventions

The experimental group was treated with TCPMs combined with conventional Western medicine, TCPMs including Xiao’er Xiaoji Zhike Oral Liquid, Pudilan Xiaoyan Oral Liquid, Lianhuaqingwen Granules, Xiaoer Feire Kechuan oral Liquid, etc. The control group was treated with Western medicine conventional treatment, including symptomatic treatment or oral macrolide drugs or intravenous infusion of macrolide drugs. We exclude RCT studies using 2 or more TCPMs or combined acupoint application and cupping therapy.

#### 2.2.4 Outcomes

The main outcome measures were fever abatement time, cough relief, or disappearance time, pulmonary rales disappearance time; secondary outcome indicators were hospitalization days, lung X-ray showed inflammatory infiltration subsided time, total effective rate, etc. The included literature all covered 1 or more of the above main indicators.

### 2.3 Database and search strategy

We will search PubMed, Web of Science, the Cochrane Library, China National Knowledge Infrastructure, Chinese BioMedical Literature, Chinese Scientific Journals Database, Embase, Clinical Trials, and Wan Fang data databases through computer to collect the RCT study of TCPMs in the treatment of children with MPP. The basic search strategy will be adjusted according to different databases.

We will search for RCTs of TCPMs in the treatment of MPP in children from the establishment of the database to October 2020 by using the combination of Medical Subject Headings words and free words. The main search terms include “Traditional Chinese patent medicine or TCPM, MPP in children, MP infection in children, MP in children, and randomized controlled trials or RCT”, etc. The detailed search strategy based on the PubMed database is shown in Table 1.

**Table 1:** Detail of the search strategy for PubMed.

NO.	Search item
1#	“Pneumonia, *Mycoplasma*” [MeSH]
2#	(((((((((((((((Pneumonia, primary atypical[title/abstract])) or (atypical pneumonia, primary [title/abstract])) or (atypical pneumonias, primary [title/abstract])) or (pneumonias, primary atypical[title/abstract])) or (primary atypical pneumonia[title/abstract])) or (primary atypical pneumonias [title/abstract])) or (*Mycoplasma* Pneumonia [title/abstract])) or (*Mycoplasma* pneumonias [title/abstract])) or (pneumonias, *Mycoplasma* [title/abstract])) or (*Mycoplasma ovipneumoniae* infection [title/abstract])) or (*Mycoplasma ovipneumoniae* infections [title/abstract])) or (*Mycoplasma pneumoniae* infection [title/abstract])) or (*Mycoplasma pneumoniae* infections [title/abstract])) or (*Mycoplasma* dispar infection [title/abstract])) or (*Mycoplasma* dispar infections[title/abstract])
3#	1# or 2#
4#	“child” [MeSH]
5#	children[title/abstract]
6#	4# or 5#
7#	“Complementary therapies” [MeSH]
8#	((((((((((((((((TCPM [Title/Abstract]) OR (traditional Chinese medicine [title/abstract])) or (therapies, complementary [title/abstract])) or (therapy, complementary [title/abstract])) or (complementary medicine [title/abstract])) or (medicine, complementary [title/abstract])) or (alternative medicine [title/abstract])) or (medicine, alternative [title/abstract])) or (alternative therapies [title/abstract])) or (therapies, alternative [title/abstract])) or (therapy, alternative [title/abstract])) or (herbal therapy [title/abstract])) or (herb therapy [title/abstract])) or (Chinese patent medicine [title/abstract])) or (Chinese proprietary medicine [title/abstract])) or (Chinese herbal drugs [title/abstract])) or (herbal [title/abstract])
9#	7# or 8#
10#	((((Randomly [title/abstract]) or (random allocation [title/abstract])) or (randomized [title/abstract])) or (controlled clinical trial [Title/Abstract])) or (randomized controlled trial [title/abstract])
11#	3# and 6# and 9#and 10#

### 2.4 Study selection and data extraction

According to the retrieval strategy formulated in advance, articles retrieved from the above database are imported into Endnote X9 document (Stanford, Connecticut, USA) management software for classified management. The 2 researchers screened the literature according to the inclusion and exclusion criteria, and cross- checked them. If there is any dispute, they will discuss it with the third researcher and solve it through negotiation. The recorded data included necessary information (title, author, journal, publication date, age, sample size, course of the disease, treatment process), specific intervention measures, detailed results.

### 2.5 Risk of bias assessment

In this study, 2 reviewers independently read the title and abstract of the literature, and further read the full text of the literature that may meet the inclusion criteria to determine whether they really meet the inclusion criteria. According to the quality evaluation standard of Cochran Library evaluation manual, the quality of methodology was evaluated from the aspects of random distribution, allocation concealment, blind method, integrity of result data, selective report, and other bias.^[27]^ The quality of the included articles is classified as “low”, “High” and “unclear”. In the evaluation process, if 2 researchers disagree, they will be resolved through mutual discussion or the third researcher will decide.

### 2.6 Statistical analysis

We will use Stata 15.0 software (LLC 4905 Lakeway drive College Station TX 77845 USA) for statistical analysis. The odds ratio is used for counting data, and the mean difference is used for measurement data. The above effect quantities are all expressed by 95% confidence interval. The results were presented in tabular form.

The inconsistency test is mainly used to evaluate the degree of inconsistency between the direct comparison results and the indirect comparison results. When there is a closed loop between various intervention measures, the inconsistency test is needed. Using Stata software for Z test, the result *P* > .05 indicates that the consistency is good, otherwise, there is inconsistency. The bias analysis of outcome indicators will carry out, and the existence of publication bias was judged according to the *P* value. If *P* > .05, there is no significant publication bias.

### 2.7 Evaluation of heterogeneity

Our meta-analysis collected the same kind of research from different countries and regions. The research design is diverse, so there are some differences. If heterogeneity exists, we will perform subgroup analysis and sensitivity analysis.

### 2.8 Subgroup analysis and sensitivity analysis

We will make a comprehensive analysis of the causes of heterogeneity. If the evidence is sufficient, we will conduct subgroup analysis to determine the source of heterogeneity. If the data is sufficient, we will consider the subgroup analysis, and conduct sensitivity analysis combined with the improvement of clinical symptoms, and further evaluate the clinical similarity and methodology included in this study to ensure the reliability of the results of this study.

### 2.9 Evaluation of publication bias

The total effective rate, the duration of fever relief, the cough relief or disappearance time, and the disappearance time of pulmonary rales after treatment were taken as the indicators. The inverted funnel plot was drawn with the standard effect amount's standard error as the ordinate and each effect quantity as the abscissa. If the inverted funnel plot is basically symmetrical, it indicates that there is a small sample effect or publication bias in this study.

### 2.10 Grading the quality of evidence

We will assess the quality of evidence regarding bias risk, indirectness, inconsistency, imprecision, and publication bias.^[28]^

## 3 Discussion

MP has become one of the most important pathogens of bronchitis and pneumonia in school-age children. The autoimmune system of school-age children is not yet perfect, the immune system is weak, and they are susceptible to MP and cross infection in school district institutions. The course of disease after MP infection is relatively long, if not timely intervention. It is easy to cause myocardial damage, liver dysfunction and other extrapulmonary manifestations, and even develop into RMPP, SMPP, which seriously damages children's health. Whether macrolide drugs, glucocorticoids, immunoglobulin, there are obvious limitations in clinical application.

TCPMs are guided by the theory of traditional Chinese medicine. The selection of prescription is rigorous and the compatibility is appropriate. After long-term clinical application, it is an effective, simple, and cheap prescription. It is processed by scientific preparation technology, with good therapeutic effect and few side effects. The dosage forms of children's medicine are mostly granules and liquid preparations, with good taste and easy to take.

In this study, we will introduce Medical Subject Headings mate analysis based on existing studies to evaluate the advantages and disadvantages of various TCPMs for the treatment of MPP in children to provide a more powerful basis for clinical rational drug use. The quality of the underlying data may bias the quality of our analysis. Therefore, in the follow-up study, we will include more high-quality, multicenter clinical, data rich studies, and evidence to evaluate the efficacy and safety of TCPMs in the treatment of MPP in children.

## Author contributions

**Conceptualization:** Hongan He, Baoqing Zhang.

**Data curation:** Hongan He, Xiao Wang.

**Methodology:** Hongan He, Jialin Zheng, Baoqing Zhang.

**Project administration:** Hongan He, Baoqing Zhang.

**Search strategy:** Hongan He, Jinjuan Wang.

**Software:** Hongan He, Yanyan Xiao.

**Statistical analysis:** Hongan He, Jialin Zheng.

**Supervision:** Jinjuan Wang.

**Writing – original draft:** Hongan He

**Writing – review & editing:** Baoqing Zhang.

## References

[1] Kumar S Roy RD Sethi GR Mycoplasma pneumoniae infection and asthma in children. Trop Doct 2019;49:117–9. 30537911 10.1177/0049475518816591

[2] Sun W To investigate the clinical characteristics and influencing factors of Mycoplasma pneumoniae infection associated with digestive system damage in children. Basic Clin Pharmacol 2019;125:27–8.

[3] Andrés-Martín A Escribano Montaner A Figuerola Mulet J Consensus Document on Community-Acquired Pneumonia in Children. SENP-SEPAR-SEIP. Arch Bronconeumol 2020;56:725–41. 32534869 10.1016/j.arbres.2020.03.025

[4] Liu WK Liu Q Chen DH Epidemiology of Acute Respiratory Infections in Children in Guangzhou: A Three-Year Study. Plos One 2014;9:e96674. 24797911 10.1371/journal.pone.0096674 PMC4010508

[5] Ozel C Dafotakis M Nikoubashman O Mycoplasma pneumoniae-induced meningoencephalitis. Fortschr Neurol Psychiatr 2015;83:392–6. 26200044 10.1055/s-0035-1553233

[6] Kutty PK Jain S Taylor TH Mycoplasma pneumoniae among children hospitalized with community-acquired pneumonia. Clin Infect Dis 2019;68:5–12. 29788037 10.1093/cid/ciy419 PMC6552676

[7] Shah SS Mycoplasma pneumoniae as a cause of community-acquired pneumonia in children. Clin Infect Dis 2019;68:13–4. 29788200 10.1093/cid/ciy421

[8] Wu QF Chen Y Zhong LM Analysis of the distribution and drug resistance of pathogens infected by Mycoplasma pneumoniae in children. Basic Clin Pharmacol 2019;125:102–3.

[9] Zhang Y Zhou Y Li S The clinical characteristics and predictors of refractory Mycoplasma pneumoniae pneumonia in children. PloS One 2016;11:e0156465. 27227519 10.1371/journal.pone.0156465 PMC4882022

[10] Yan C Xue G Zhao H Molecular and clinical characteristics of severe Mycoplasma pneumoniae pneumonia in children. Pediatr Pulmonol 2019;54:1012–21. 31119869 10.1002/ppul.24327

[11] He J Liu M Ye Z [Corrigendum] Insights into the pathogenesis of Mycoplasma pneumoniae (Review). Mol Med Rep 2018;17:4155–6. 29286101 10.3892/mmr.2017.8324 PMC5802185

[12] Li SL Xue GH Zhao HQ The Mycoplasma pneumoniae HapE alters the cytokine profile and growth of human bronchial epithelial cells. Bioscience Rep 2019;39:BSR20182201. 10.1042/BSR20182201 PMC6340952 30573530

[13] Shi S Zhang X Zhou Y Immunosuppression Reduces Lung Injury Caused by Mycoplasma pneumoniae Infection. Sci Rep 2019;9:7147–56. 31073201 10.1038/s41598-019-43451-9 PMC6509254

[14] Yang MY Meng FZ Gao M Cytokine signatures associate with disease severity in children with Mycoplasma pneumoniae pneumonia. Sci Rep-Uk 2019;9:17853–64. 10.1038/s41598-019-54313-9 PMC6882793 31780733

[15] Bodhankar S Sun X Woolard MD Interferon gamma and interleukin 4 have contrasting effects on immunopathology and the development of protective adaptive immunity against mycoplasma respiratory disease. J Infect Dis 2010;202:39–51. 20504237 10.1086/653121 PMC4086782

[16] Dumke R Jacobs E Antibody response to Mycoplasma pneumoniae: protection of host and influence on outbreaks? Front Microbiol 2016;7:39–45. 26858711 10.3389/fmicb.2016.00039 PMC4726802

[17] Youn YS Lee KY Mycoplasma pneumoniae pneumonia in children. Korean J Pediatr 2012;55:42–7. 22375148 10.3345/kjp.2012.55.2.42 PMC3286761

[18] Ha SG Oh KJ Ko KP Therapeutic efficacy and safety of prolonged macrolide, corticosteroid, doxycycline, and levofloxacin against macrolide-unresponsive Mycoplasma pneumoniae pneumonia in children. J Korean Med Sci 2018;33:e268. 30344461 10.3346/jkms.2018.33.e268 PMC6193889

[19] Yilmaz C Ozcengiz G Antibiotics: pharmacokinetics, toxicity, resistance and multidrug efflux pumps. Biochem Pharmacol 2017;133:43–62. 27765485 10.1016/j.bcp.2016.10.005

[20] Mocanu A Ivanov A Alecsa M Uncommon erythema multiforme in small children: experience of a single Romanian pediatric unit two case reports. Medicine 2019;98:e17895. 31725635 10.1097/MD.0000000000017895 PMC6867757

[21] Pereyre S Goret J Bebear C Mycoplasma pneumoniae: Current Knowledge on Macrolide Resistance and Treatment. Front Microbiol 2016;7:974–84. 27446015 10.3389/fmicb.2016.00974 PMC4916212

[22] Varghese SM Kerkar VV Macrolide Resistant Mycoplasma pneumoniae in Community Acquired Pneumonia. Indian J Pediatr 2020;87:1–2. 32333367 10.1007/s12098-020-03301-3

[23] Chen YC Hsu WY Chang TH Macrolide-resistant Mycoplasma pneumoniae infections in pediatric community-acquired pneumonia. Emerg Infect Dis 2020;26:1382–91. 32568052 10.3201/eid2607.200017 PMC7323531

[24] Yang HJ Song DJ Shim JY Mechanism of resistance acquisition and treatment of macrolide-resistant Mycoplasma pneumoniae pneumonia in children. Korean J Pediatr 2017;60:167–74. 28690643 10.3345/kjp.2017.60.6.167 PMC5500384

[25] Shamseer L Moher D Clarke M Preferred reporting items for systematic review and meta-analysis protocols (PRISMA-P) 2015: elaboration and explanation. BMJ 2015;350:g7647. 25555855 10.1136/bmj.g7647

[26] Nelson JD Community-acquired pneumonia in children: guidelines for treatment. Pediatr Infect Dis J 2000;19:251–3. 10749470 10.1097/00006454-200003000-00016

[27] Higgins JP Altman DG Gotzsche PC The Cochrane Collaboration's tool for assessing risk of bias in randomised trials. BMJ 2011;343:d5928. 22008217 10.1136/bmj.d5928 PMC3196245

[28] Puhan MA Schunemann HJ Murad MH A GRADE Working Group approach for rating the quality of treatment effect estimates from network meta-analysis. BMJ 2014;349:g5630. 25252733 10.1136/bmj.g5630

